# A new species of the ant genus *Lasius* Fabricius, 1804 from Crete (Hymenoptera, Formicidae)

**DOI:** 10.3897/zookeys.789.27022

**Published:** 2018-10-10

**Authors:** Sebastian Salata, Lech Borowiec

**Affiliations:** 1 Department of Biodiversity and Evolutionary Taxonomy, University of Wrocław, Przybyszewskiego 65, 51-148 Wrocław, Poland University of Wrocław Wrocław Poland

**Keywords:** Endemic species, Greece, *
Lasius
*, Mediterranean

## Abstract

*Lasiustapinomoides***sp. n.** from Crete, Greece, is described and illustrated. It belongs to *L.turcicus* complex and is well characterized by very small body, extremely shallow metanotal groove and presence of suberect to erect setae on the apical part of scape. New records of Cretan members of the genus *Lasius* Fabricius, 1804 are provided, their checklist is updated, and the key to their determination is presented.

## Introduction

The genus *Lasius* Fabricius, 1804 is widely distributed throughout the Holarctic. Within its range, it is one of the most abundant of all Formicidae genera and its species are very often dominants of local myrmecofauna (Janda et al. 2004). Forty-three *Lasius* species are known from Europe and Mediterranean area (Borowiec 2014, Talavera et al. 2015, Seifert and Galkowski 2016), divided in five subgenera (Maruyama et al. 2008). The most numerous is the nominotypical subgenus Lasius s. str. Ruzsky, 1913 – 23 species, followed by *Chthonolasius* Ruzsky, 1913-13 species, *Cautolasius* Wilson, 1955 – 3 species, *Dendrolasius* Ruzsky, 1913 – 2 species, and *Austrolasius* Faber, 1967 - 2 species (Borowiec 2014, Talavera et al. 2015, Seifert and Galkowski 2016). Most of these species are characterised by possessing a wide geographical range. Only a few representatives of the subgenus Lasius s. str. are known from narrow area and could be considered as endemic species. Among these are *Lasiuskarpinisi* Seifert, 1992, known from Mt. Timfristos in the Greek mainland, *Lasiusbalearicus* Talavera, Espadaler & Vila, 2015 described from Mallorca and *Lasiuscasevitzi* Seifert & Galkowski, 2016 inhabiting Corsica.

Crete, as one of the largest Mediterranean islands, with very diverse, mountainous landscape (Grove and Rackham 1993), has poorly known myrmecofauna and requires more detailed study. Based on the literature, seven *Lasius* species are known from this island (Forel 1886, 1889, 1910, Neuenschwander et al. 1983, Legakis 2011, Borowiec and Salata 2012). However, presence of some of them requires confirmation. During our fieldwork, performed in different parts of Crete, new ant material was collected. Together with material deposited in the Natural History Museum of Crete it was used to review Cretan *Lasius* species. As results of this research a new species has been discovered and few other species have been recognised as new records for Crete. Below we describe *Lasiustapinomoides*, a new Cretan endemic, provide detailed occurrence data of other Cretan *Lasius* species, and present an identification key.

## Materials and methods

Ants were sampled between 2007 and 2014 from sites in different parts of Crete. The method was direct sampling (hand collecting). Individual specimens and nests were collected on the ground, in leaf litter and rock rubble, under stones and tree trunks. All specimens were preserved in 75% EtOH. Study was supported with material deposited in the Natural History Museum of Crete (Iraklion, Greece).

Examined specimens are housed in the following collections:

**BMNH**Natural History Museum, London;

**DBET** Department of Biodiversity and Evolutionary Taxonomy, University of Wroclaw, Poland;

**HNHM**Hungarian National History Museum, Budapest, Hungary;

**MSNG**Museo Civico di Storia Naturale, Genova, Italy;

**NHMB**Naturhistorisches Museum, Basel;

**NHMC**Natural History Museum of Crete, Iraklion;

**SMNG**Senckenberg Museum für Naturkunde Görlitz, Görlitz, Germany.

Specimens were compared using standard methods of comparative morphology. Photos were taken using a Nikon SMZ 1500 stereomicroscope, Nikon D5200 photo camera, and Helicon Focus software.

All given label data are in original spelling, presented in quotation marks; a slash (/) separates data on different rows and double slash (//) separate labels.

Specimens of *Lasiustapinomoides* sp. n. were compared with all other known Cretan species of the genus *Lasius* and type material of members of the *Lasiusalienus* group listed below. Type specimens photographs of the *Lasiusalienus* group members are available online on AntWeb (www.AntWeb.org) and are accessible using the unique CASENT or FOCOL identifying specimen code. Moreover, we compared them with samples of members of the *Lasiusalienus* group from other Greek regions. Data concerning distribution of Greek *Lasius* samples used in the comparison is provided in series of regional checklists (Borowiec and Salata 2012, 2013, 2014, 2017a,b, 2018, Bračko et al. 2016). Therefore, we see no reason to repeat this information. The list of Cretan *Lasius* species, together with their occurrence data on the island is provided below. This study was also supported by data published in recent revisions of *Lasius* s. str. (Seifert 1992, Talavera et al. 2015, Seifert and Galkowski 2016).

We decided to list all other ant species collected from the same localities where the new species has been found. In our opinion it provides valuable information about ecosystem structure and species diversity characteristic for habitats preferred by this species. Distribution maps of all recorded *Lasius* species were created in DivaGis 7.5 (Hijmans et al. 2011).

Measurements:

**EL** eye length; measured along the maximum vertical diameter of eye;

**EW** eye width; measured along the maximum horizontal diameter of eye;

**HL** head length; measured in straight line from mid-point of anterior clypeal margin to mid-point of posterior margin; the head must be carefully tilted to the position with the true maximum; excavations of posterior margin reduce HL;

**HTL** hind tibia length; maximum length of hind tibia;

**HW** head width; measured in full-face view directly above the eyes;

**ML** mesosoma length; measured as diagonal length from the anterior end of the neck shield to the posterior margin of the propodeal lobe;

**PNW** pronotum width; maximum width of pronotum in dorsal view;

**SL** maximum straight-line scape length excluding the articular condyle.

Indices:

**HI**HW/HL * 100;

**SI1**SL/HL * 100;

**SI2**SL/HW * 100;

**MI**HTL/ML * 100;

**EI1**EW/EL * 100;

**EI2**EW/HL * 100;

**TI**HW/HTL * 100.

Abbreviations:

**q**. gyne;

**w**. worker.

Pilosity inclination degree applies to this used in Hölldobler and Wilson (1990). The adpressed (0–5°) hairs run parallel, or nearly parallel to the body surface. Decumbent hairs stand 10–15°, subdecumbent hair stands 30°, suberect hairs stand 35–45°, the erect hairs stand more than 45° from the body surface.

### Type material of taxa compared with *Lasiustapinomoides* sp. n.

*Lasiusalienus* (Foerster, 1850), neotype (w.) (FOCOL0754): ‘‘GER: Eifel, 7.9.1991 / 37km SE Aachen / Schleiden / leg. Seifert // *Formicaaliena* / Förtser 1850 / Neotype / des B. Seifert 1992’’ (SMNG);

*L.austriacus* Schlick-Steiner, Steiner, Schödl & Seifert, 2003, paratype (w.) (CASENT0916646): “#11055: Feldberg near / Pulkau, Austria (15°51’E/ / 48°40’N), 360 m / a.s.l., 06.08.2002. // leg. B.C. Schlick-Steiner & / F.M Steiner // *Lasiusaustriacus* / Schlick-Steiner / 2003 / PARATYPE” (HNHM); paratype (q.) (CASENT0916647): “#11055: Feldberg near / Pulkau, Austria (15°51’E/ / 48°40’N), 360 m / a.s.l., 06.08.2002. // PARATYPE / *Lasiusaustriacus* / design. Schlick-Steiner, Steiner / Schödl & Seifert 2003” (HNHM);

*L.neglectus* Van Loon, Boomsma & Andrasfalvy, 1990, paratype (w.) (CASENT0903220): ‘‘*Lasiusneglectus* // HUNGARY / Budapest / 1.VII.88 / JJ. Boomsma // ANTWEB / CASENT / 0903220 // BMNH(E) / 1016243 // PARA- / TYPE’’ (BMNH);

*Lasiusparalienus* Seifert, 1992, paratype (w.) (FOCOL0751): ‘‘Germania: Kr. Bautzen / 2 km S Weißenberg; N066 / 11.7.1991, leg. Seifert // *Lasiusparalienus* / Seifert / Holotypus’’ (SMNG);

*Lasiuspsammophilus* Seifert, 1992, holotype (w.) (FOCOL0752): ‘‘GER: Kr Weißwasser / 4 km N Steinbach: N 135 / 30.7.1991 leg. Seifert // *Lasiuspsammophilus* / Seifert / Holotype’’ (SMNG);

*Lasiusturcicus* Santschi, 1921, lectotype (w.) (CASENT0912297): ‘‘*Lasius* / turcicus / Santschi / Type / SANTSCHI det. 1920 // lectotype / desig. by / E. O. Wilson // Asie min. / Angora / G. d. Kerville // Sammlung / Dr. F. Santschi / Kairouan // ANTWEB / CASENT / 0912297’’ (NHMB).

## Results

### Lasius tapinomoides sp. n.

Taxon classificationAnimaliaHymenopteraFormicidae

http://zoobank.org/412DD1F4-21DA-4A85-B4CB-5031FC049560

**Figures 1–2.**
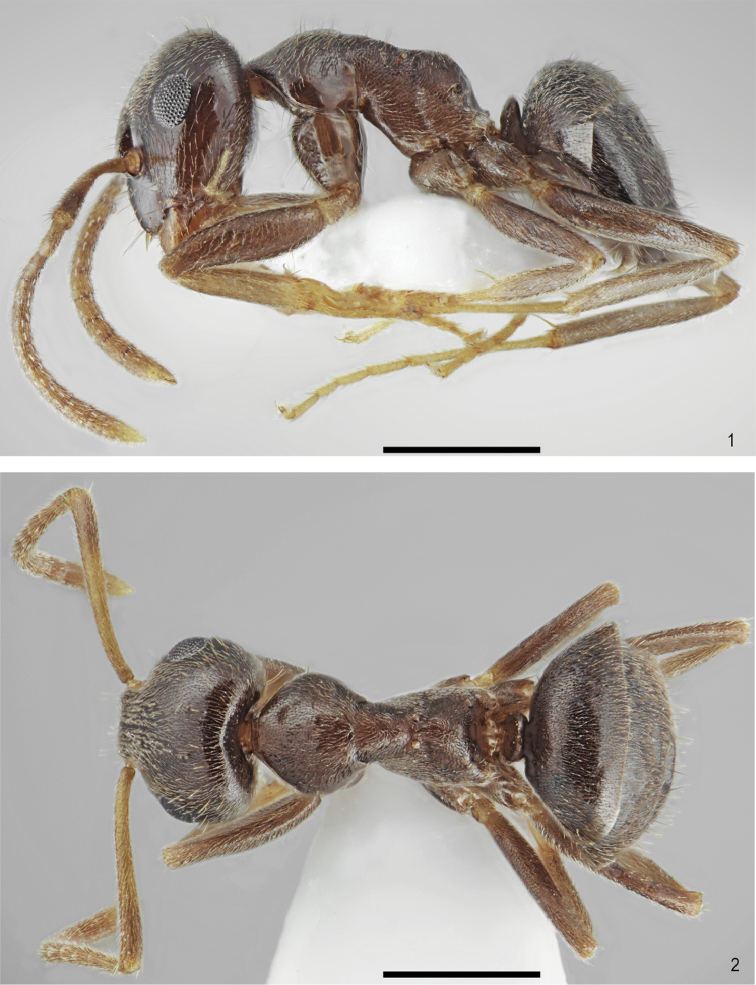
*Lasiustapinomoides* sp. n., holotype. **1** Lateral view **2** Dorsal view. Scale bar: 0.5 mm.

**Figures 3–5.**
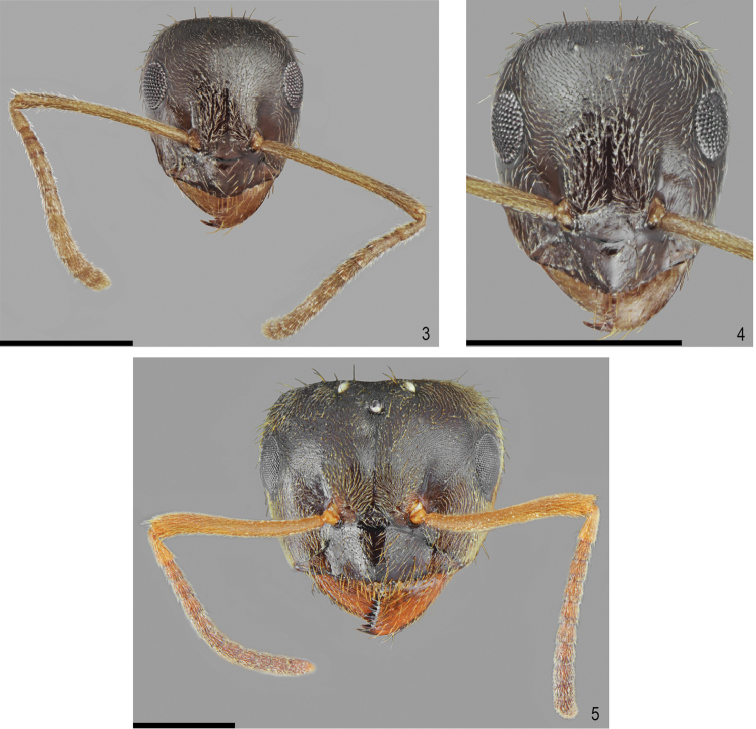
*Lasiustapinomoides* sp. n. **3** Holotype, head with antennae **4** Holotype, head **5** Gyne, head. Scale bar: 0.5 mm.

**Figures 6–7.**
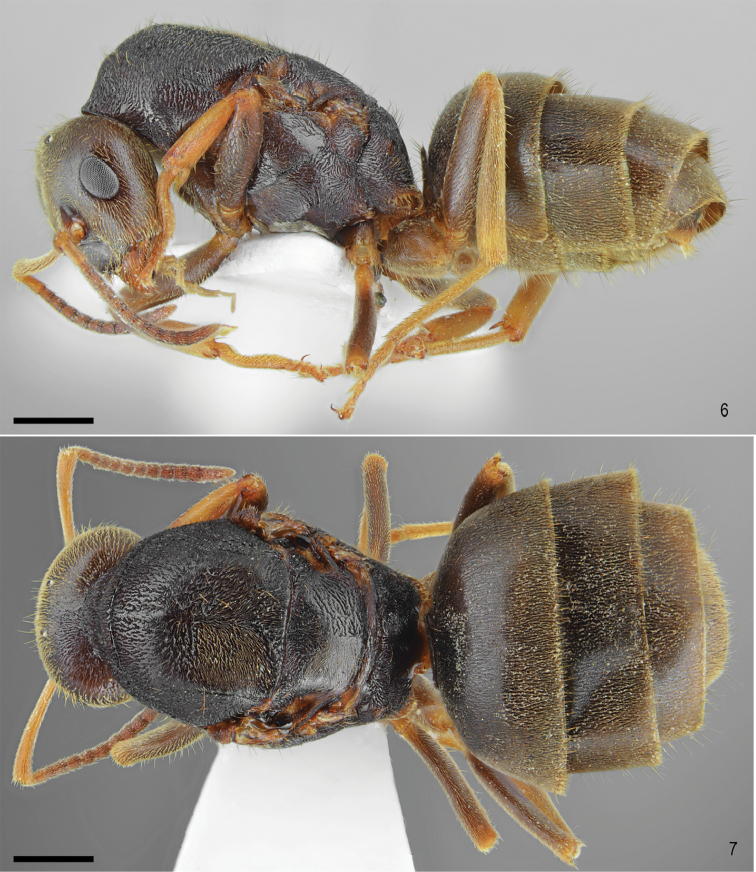
*Lasiustapinomoides* sp. n., gyne. **6** Lateral view **7** Dorsal view. Scale bar: 0.5 mm

Figs 1–2 , 3–5 , 6–7

#### Type material.

Holotype (w.): ‘‘*Lasius* / *tapinomoides* sp. nov. / HOLOTYPE // Collection L. Borowiec / Formicidae / LBC-GR00976 // GREECE, Crete, Rethymno Pr. / Antonios Spilia Gorge / 35°15.245 N,24°34.220 E / 11 V 2013, 342 m / L.Borowiec // CASENT0845075’’ (DBET); paratypes (6w.,1q.): data same as holotype, CASENT0845076 to CASENT0845082 (DBET, NHMC, NHMB); paratypes (15w.), CASENT0845460 to CASENT0845474: ‘‘GREECE, Crete, Rethymno Pr. / Kato Malaki / 35.28333 N,24.4 E / 15 V 2013, 235 m / L. Borowiec’’ (DBET, NHMC, BMNH).

#### Non-type material.

2w. (pin): ‘‘Collection L. Borowiec / Formicidae / LBC-GR00467 // GREECE, W Crete, 339 m / Plemeniana n. Kandanos / 35.31666 N,23.71666 E / 2 V 2011, L. Borowiec (DBET); 1w. (pin): Collection L. Borowiec / Formicidae / LBC-GR01420 // GREECE, Crete, Rethymno / Orthes Gorge, 318 m / 35.3336 N,24.6848 E / 28 IV 2014, S. Salata’’ (DBET); 2w. (pin): ‘‘Collection L. Borowiec / Formicidae / LBC-GR00993 // GREECE, Crete, Rethymno / n. Argioupolis / 35.28333 N,24.33333 E / 13 V 2013, 197 m / L. Borowiec’’ (DBET); 1w. (pin): ‘‘GREECE, Crete, Rethymno Pr. / Preveli Beach / 35.16666 N,24.46666 E / 7 V 2013, 10 m / L. Borowiec’’ (DBET); 7w. (pin): ‘‘GREECE, Crete, Rethymno Pr. / Road to Preveli Beach loc. 1 / 35.16666 N,24.45 E / 7 V 2013, 58 m / L. Borowiec’’ (DBET; 12w. (EtOH), Crete, Lasithi Prov., Gorge of Richtis, 35.16667N, 25.98333E, 245m, 08.iv.2014, leg. S. Salata (DBET); 2w. (EtOH), Orthes Gorge, 35.3336N,24.6848E, 318 m, 28.iv.2014, leg. S. Salata (DBET); 3w. (EtOH), Antonios Spilia Gorge, 35°15.245 N,24°34.220, 342m, 11.v.2013, leg. S. Salata (DBET); 5w. (EtOH), road to Preveli Beach loc. 1, 35.16666N,24.45E, 58m, 7.v.2013, leg. S. Salata (DBET).

#### Etymology.

The name refers to the similarity of this species to species of the *Tapinoma* genus, caused by a very shallow metanotal groove.

#### Description.

Worker.

Measurements: see Table 1.

Head, mesosoma, petiole and gaster uniformly coloured, brown to dark brown. Antennae, tibiae and tarsi bright brown to orange (Figs 1–4).

**Table 1.** Measurements and indices: *L.tapinomoides* and *L.turcicus*.

Measurements and indices	*L.tapinomoides* N = 10	*L.turcicus* N = 10
HL	0.707 ± 0.02 (0.679–0.749)	0.891 ± 0.06 (0.782–0.983)
HW	0.578 ± 0.03 (0.525–0.637)	0.798 ± 0.06 (0.682–0.905)
SL	0.668 ± 0.02 (0.625–0.715)	0.836 ± 0.04 (0.743–0.95)
EL	0.179 ± 0.008 (0.167–0.19)	0.214 ± 0.012 (0.201–0.235)
EW	0.132 ± 0.004 (0.123–0.136)	0.157 ± 0.015 (0.123–0.184)
ML	0.791 ± 0.04 (0.726–0.827)	1.06 ± 0.08 (0.935–1.18)
HTL	0.709 ± 0.02 (0.682–0.76)	0.892 ± 0.05 (0.799–0.961)
PNW	0.417 ± 0.018 (0.38–0.447)	0.558 ± 0.04 (0.48–0.631)
HI	81.8 ± 3.3 (73.4–85.0)	90.2 ± 1.8 (87.2–92.8)
SI1	94.5 ± 1.5 (91.4–96.3)	93.9 ± 2.9 (87.8–98.7)
SI2	115.7 ± 4.4 (112.2–127.6)	104.7 ± 3.6 (97.3–109.3)
MI	89.0 ± 3.1 (84.8–95.5)	84.5 ± 3.0 (81.1–91.9)
EI1	74.0 ± 3.6 (68.7–80.2)	73.3 ± 4.6 (61.2–78.3)
EI2	18.9 ± 0.9 (17.0–19.9)	17.8 ± 1.3 (15.0–19.8)
TI	82.0 ± 2.6 (75.8–85.2)	89.2 ± 2.2 (85.4–93.8)

Head oval, 1.2 times as wide as long, lateral surfaces above eyes convex, occipital margin of head slightly convex (Figs 3–4). Clypeus shiny and smooth, its anterior margin convex, lacking median anterior notch, covered with sparse, decumbent to erect pubescence, average distance between setae longer than three fourths of their length. Masticatory border of mandibles with 7–8 teeth. Eyes medium-sized, oval, 0.25 times as long as length of the head. Antennal scape long, straight or slightly curved on its anterior part, 0.9 times as long as length of the head, exceeding beyond occipital margin of head, in apex gradually widened. Pedicel more than 2.0 times longer than wide, average 2.5 times longer than second segment of funiculus. Other funicular segments from 1.5 to 2.0 times longer than wide (Figs 3–4). Surface of scape with very fine microreticulation, shiny. Its surface covered with thin, dense, adpressed setae, on its apical part several suberect setae also occur.

Genae with few adpressed to suberect setae (Figure 3). Underside of head with thin, dense, adpressed setae and a few long, suberect to erect setae (Figure 1). Whole frontal head surface covered with short, adpressed and dense pubescence and sparse, long, thick suberect to erect setae, the distance between setae at least as long as three fourths of their length (Figs 3–4).

Mesosoma short, 1.9 times as long as wide. In lateral view, promesonotum low and flattened, metanotal groove very shallow, propodeum very low, propodeal dorsum slightly convex, propodeal declivity convex, less than twice length of propodeal dorsum (Figure 1). Whole mesosoma surface shiny with sparse microreticulation, covered with short, adpressed and dense pubescence, and sparse, long, thick suberect to erect setae, the distance between setae at least as long as half of their length, metapleuron below the level of the propodeal spiracle with more than 5 setae (Figs 1–2).

Petiole scale low, in lateral view with slightly convex sides, its dorsal crest thick and arched (Figure 1). Gaster with very rare and fine microreticulation, shiny, bearing pilosity similar to this covering mesosoma. Legs long, shiny, with fine microreticulation. Surface of tibia and femora with thin, dense, adpressed to subdecumbent setae, extensor profile without erect setae (Figs 1–2).

#### Description.

Gyne.

Measurements and Indices (n=1): HL: 1.03; HW: 1.2; SL: 0.9; EL: 0.3; EW: 0.24; ML: 2.3; HTL: 1.3; PNW: 1.4; HI: 116.5; SI1: 87.4; SI2: 75; MI: 56.5; EI1: 80; EI2:23; TI: 92.

Head, mesosoma, petiole and gaster dark brown. Antennae, tibiae and tarsi bright brown to orange (Figs 5–7).

Head trapezoidal, 1.1 times as wide as long, lateral surfaces above eyes convex, sides of occipital margin of head slightly convex, its central part concave (Figure 5). Clypeus shiny and smooth, its anterior margin convex, lacking median anterior notch, covered with decumbent to erect setae, average distance between setae longer than one third of their length. Masticatory border of mandibles with seven teeth. Eyes medium-sized, oval, 0.3 times as long as length of the head. Antennal scape short, curved on its anterior part, 0.9 times as long as length of the head, slightly exceeding beyond occipital margin of head, in apex gradually widened. Pedicel more than 2.0 times longer than wide, 2.0 times longer than second segment of funiculus. Other funicular segments from 1.5 to 2.0 times longer than wide (Figure 5). Surface of scape with very fine microreticulation, shiny. Its surface covered with thin, dense, adpressed to decumbent pubescence, on its apical part a few short, suberect setae also occur. Genae with thin, dense adpressed pubescence and a few suberect setae (Figure 5). Underside of head with thin, dense, adpressed pubescence and a few long, suberect setae (Figure 6). Whole frontal head surface covered with short, adpressed and dense pubescence and sparse, long, thick suberect to erect setae, the distance between setae at least as long as three fourths of their length (Figs 5–6).

Mesosoma long, 1.6 times as long as wide. In lateral view moderately high, its dorsum slightly convex, propodeal dorsum slightly convex, propodeal declivity convex (Figure 6). Whole mesosoma surface shiny with very sparse microreticulation, covered with short, adpressed and dense pubescence, and sparse, long, thick suberect to erect setae, the distance between setae at least as long as half of their length (Figs 6–7).

Petiole scale low and wide, in lateral view with slightly convex sides, its dorsal crest wide and deeply concave in central part. Gaster with moderately thick and fine microreticulation, shiny, bearing pilosity denser than this covering mesosoma. Legs long, shiny, with fine microreticulation. Surface of tibia and femora with thin, dense, adpressed to subdecumbent setae, extensor profile of tibia with erect setae (Figs 6–7).

#### Differential diagnosis.

Worker. As a member of the *L.alienus* group it is characterized by dorsal plane of scape, genae, and extensor profile of hind tibiae lacking or having very few erect or suberect setae and, in all species known from Crete, presence of >15 erect setae on the occipital edge of the head. Within the *L.alienus* group it can be classified to the *L.turcicus* complex. This complex can be characterized by small number of mandibular teeth (6–8), usually lack of suberect setae on hind tibia, very sparse clypeal pubescence, and more or less shallow metanotal groove. There are three known species of this complex: *L.turcicus*, *L.neglectus* and *L.austriacus*. *Lasiustapinomoides* sp. n. differs from all of them in presence of suberect to erect setae on antennal scape covering its apical part (ca. 1/3 upper part of the scape). Additionally from first two relatives it differs also in very shallow metanotal groove and from *L.austriacus* it differs in more flattened promesonotum, antennal sockets set not very close to posterior clypeal margin and habitat preferences. *Lasiusaustriacus* is related with xerothermous sites (Schlick-Steiner et al. 2003, Steiner et al. 2004) while *L.tapinomoides* inhabits moist, closed canopy forests.

There are two other species of the *L.alienus* group known from Crete: *L.bombycina* Seifert & Galkowski, 2016 and *L.turcicus* Santschi, 1921. *Lasiustapinomoides* differs from all of them in very small body size. Nevertheless, at the first glance it can be confused with small workers of *L.turcicus*, from which it differs in the following measurements (*L.tapinomoides* sp. n. vs *L.turcicus*): HI: 81.8 ± 3.3 (73.4–85.0) vs 90.2 ± 1.8 (87.2–92.8), SI2: 115.7 ± 4.4 (112.2–127.6) vs 104.7 ± 3.6 (97.3–109.3), TI: 82.0 ± 2.6 (75.8–85.2) vs TI: 89.2 ± 2.2 (85.4–93.8). For more measurements data see Table 1.

Gyne. During our fieldwork we could observe several gynes of *L.tapinomoides* and all detected nests were monogynous. Unfortunately, we were able to collect only a single specimen, therefore we provide very scarce data. As a coherent differential diagnosis is impeded, we decided to limit it to the most visible difference. Based on the morphology, *L.tapinomoides* differs from *L.turcicus*, *L.austriacus* and *L.neglectus* in presence of erect setae on tibiae.

#### General distribution.

Greece: Crete – endemic species.

#### Biology.

Species inhabiting moist, closed canopy forests, which are most often located in stream valleys. Nesting in wet soil, under shallow and small rocks. Nests, most often, located in the vicinity of water sources. Workers were found in the litter or on the rocks surrounding the nest entrance. Colonies monogynous.

The following ant species were recorded in the same areas as *L.tapinomoides*:

Antonios Spilia Gorge: *Aphaenogastercecconii* Emery, *A.rugosoferruginea* Forel, *A.simonellii* Emery, *Camponotuscandiotes* Emery, *C.lateralis* (Olivier), *Crematogasterionia* Forel, *Messorwasmanni* Krausse, Pheidolecf.pallidula, *Stigmatommadenticulatum* Roger, *Temnothoraxariadnae* Csősz, Heinze & Mikó, T.cf.graecus, T.cf.exilis, T.cf.luteus, *Tetramoriumcaespitum* (Linnaeus), *T.diomedeum* Emery;

Plemeniana n. Kandanos: *Aphaenogasterrugosoferruginea*, *A.simonellii*, *Camponotuscandiotes*, *C.gestroi* Emery, *C.lateralis*, *Colobopsistruncata* (Spinola), Crematogastercf.ionia, *Lasiuslasioides* (Emery), *Messoribericus* Santschi, *Tetramoriumcaespitum*;

Orthes Gorge: *Aphaenogastercecconii*, A.cf.subterranea, *A.simonellii*, *Camponotusbaldaccii* Emery, *C.candiotes*, *C.jaliensis* Dalla Torre, *C.kiesenwetteri* (Roger), *C.rebeccae* Forel, *Crematogastersordidula* (Nylander), *Lasiuslasioides*, *Lepisiotanigra* Dalla Torre, Messorcf.muticus, *M.wasmanni*, Tetramoriumcf.caespitum, *T.kephalosi* Salata & Borowiec

n. Argioupolis: *Aphaenogastercecconii*, *A.rugosoferruginea*, *A.simonellii*, *Camponotuscandiotes*, *C.lateralis*, Crematogastercf.ionia, *Lepisiotanigra*, Messorcf.muticus, *M.wasmanni*, Pheidolecf.pallidula, *Plagiolepispallescens* sensu Radchenko, Temnothoraxcf.luteus, *T.ariadnae*, T.cf.exilis, T.cf.graecus, Tetramoriumcf.caespitum;

Preveli Beach: *Aphaenogastercecconii*, *Crematogasterionia*, *Lasiuspsammophilus* Seifert, *Lepisiotamelas* (Emery), Pheidolecf.pallidula, *Tapinomafestae* Emery, *Tetramoriumkephalosi*;

road to Preveli Beach loc. 1: *Aphaenogasterrugosoferruginea*, *A.simonellii*, *Camponotusbaldaccii*, *C.candiotes*, *C.gestroi*, *C.kiesenwetteri*, *C. lateralis*, *Cardiocondylamauritanica* Forel, Crematogastercf.ionia, *Cryptoponeochracea* (Mayr), *Lasiuslasioides*, *L.bombycina* Seifert & Galkowski, *L.psammophilus*, *Lepisiotanigra*, *Messoribericus*, *M.wasmanni*, Pheidolecf.pallidula, *Plagiolepispallescens* sensu Radchenko, Temnothoraxcf.luteus, T.cf.graecus, *T.recedens* (Nylander), Tetramoriumcf.caespitum;

Kato Malaki: *Aphaenogastersimonellii*, *Camponotusbaldaccii*, *C.gestroi*, *C.jaliensis*, Crematogastercf.ionia, *Crematogastersordidula*, *Messoribericus*, *M.wasmanni*, Pheidolecf.pallidula, *Plagiolepispallescens* sensu Radchenko, *Temnothoraxariadnae*, *T.recedens*, *Tetramoriumdiomedeum*, *Tetramoriumpunctatum* Santschi;

Gorge of Richtis: *Aphaenogasterrugosoferruginea*, *Camponotuscandiotes*, *C.gestroi*, *C.lateralis*, *Crematogastersordidula*, *Lasiuslasioides*, *Messorwasmanni*, Temnothoraxcf.exilis, T.cf.graecus, Tetramoriumcf.caespitum.

#### Comments.

We examined several hundred specimens of *Lasiusturcicus* from 76 samples across Greece and western Turkey (including initial nest samples), also numerous samples from Crete (see new material listed below). In none of these samples did we find specimens with such a shallow metanotal groove like in *L.tapinomoides*. This character is constant in all examined specimens and is always correlated with very small body size of workers and preference to humid habitats. Across all the sampled area within Greece and Turkey, where we collected species of the *L.turcicus* complex, we have not found workers or nests with workers similar to *L.tapinomoides*. Moreover, *L.tapinomoides* is the only known member of the *L.turcicus* complex that have suberect to erect setae on the apical 1/3 part of the antennal scape. This has prompted us to hypothesize that Cretan samples represent a new species.

##### New records of Cretan members of the genus *Lasius*

***Lasiusbombycina* Seifert & Galkowski, 2016**

**GREECE**, **Crete**: 2w (EtOH), Omalos, 35.31667N,23.9E, 1122 m, 03.v.2014, leg. S. Salata (DBET); 4w. (pin), 03.v.2011, Omalos Plateau, 35.33333N,23.88333E, 1034 m, leg. L. Borowiec (DBET); 1w. (EtOH), Potamida n. Mythimna, 35.46666N,23.68333E, 37 m, 02.v.2011, leg. L. Borowiec (DBET); 1w. (pin), W of Georgioupoli, 35.36666N,24.25E, 17 m, 02.v.2007, leg. L. Borowiec (DBET); 8w. (EtOH), Zaros Lake, 35.13333N,24.9E, 409 m, 26.iv.2014, leg. S. Salata (DBET); 3w. (EtOH), Kato Symi loc. 3, 35.05N,25.48333E, 818 m, 16.iv.2014, leg. S. Salata (DBET); 4w. (EtOH), Lasithi Platou – Plati, 35.16667N,25.43333E, 831 m, 01.v.2014, leg. S. Salata (DBET); 2w. (EtOH), Orino, 35.06667N,25.9E, 523 m, 12.iv.2014, leg. S. Salata (DBET); 2w. (pin), Ag. Joannis Forest loc. 1, 35.23333N,24.4E, 448 m, 06.v.2013, leg. L. Borowiec (DBET); 1w. (pin), Ampelakiou, 35.26666N,24.46666E, 464 m, 10.v.2013, leg. L. Borowiec (DBET); 1w. (EtOH), Fourfouras, 35.21666N,24.71666E, 578 m, 14.v.2013, leg. S. Salata (DBET); 5w. (EtOH), n. Velonado, 35.25N,24.36667E, 373 m, 13.v.2013, leg. S. Salata (DBET); 2w. (EtOH), n. Vilandredo, 35.25N,24.31667E, 354 m, 13.v.2013, leg. S. Salata (DBET); 2w. (EtOH), road to Preveli Beach loc. 1, 35.16666N,24.45E, 58 m, 7.v.2013, leg. S. Salata (DBET); 3w. (EtOH), Selli-Oros rd., 35.28333N,24.5E, 473 m, 11.v.2013, leg. S. Salata, (DBET); 1w. (pin), Spili-Gerakari rd. loc. 2, 35.21987N,24.57144E, 804 m, 09.v.2013, leg. L. Borowiec (DBET); 1w. (EtOH), Asi Gonia, 35.25N,24.26667E, 716 m, 29.v.2001, leg. S. Salata (DBET); 1w. (EtOH), Niato plateau, 35.28333N,24.13333E, 1200 m, 19.vii.2013, leg. S. Salata (DBET); 1w. (EtOH), Therisso to Kaloros Mt., 35.35N,23.95E, 1130 m., 31.xii.2013, leg. Simaiakis (NHMC); 1w. (EtOH), Therisso to Kaloros Mt., 35.3667N,23.9833E, 1134 m., 19.Vii.2013, leg. Simaiakis (NHMC); 1w. (EtOH), Diplori, 35.1667N,24.9333E, 1350 m., 19.x.1999, leg. Nikolakakis (NHMC); 1w. (EtOH), Dikti Mt., 35.11667N,25.4667E, 1450 m., 9.i.2001, leg. Simaiakis (NHMC); 1w. (EtOH), Tigania, 35.28333N,24.7333E, 1100 m., 7.iv.2000, leg. Nikolakakis (NHMC).

***Lasiusmyops* Forel, 1894**

**GREECE**, **Crete**: 3w. (pin), 3w. (EtOH), Anopoli, 35.26667N,24.06667E, 1780 m, 22.vii.2006, leg. Chatzaki M. (NHMC, DBET).

***Lasiusillyricus* Zimmermann, 1935**

**GREECE**, **Crete**: 6w. (pin), Limnakarou platou, 35.13333N,25.46667E, 1130 m, 26.iv.2014, leg. S. Salata (DBET); 6w. (EtOH), Limnakarou platou, 35.13333N,25.46667E, 1130 m, 10.iv.2014, leg. S. Salata (DBET); 2w. (EtOH), Dikti mt., 35.1N,25.46667E, 1750 m, 05.viii.2000, leg. M. Chatzaki (NHMC).

***Lasiuslasioides* (Emery, 1869)**

**GREECE**, **Crete**: 1w. (pin), Agia, 6 km SW Chania, 35.46666N,23.91666E, 22 m, 03.v.2011, leg. L. Borowiec (DBET); 5w. (pin), Askifou, 35.26666N,24.16666E, 730 m, 01.v.2007, leg. L. Borowiec & M.L. Borowiec (DBET); 7w. (EtOH), Diktamos Gorge n. Stilos, 35.43333N,24.1E, 160 m, 04.v.2011, leg. L. Borowiec (DBET); 3w. (EtOH), Kalives river, 35.45N,24.13333E, 26 m, 01.v.2014, leg. S. Salata (DBET); 1w. (pin), Kourna Lake, 35.31666N,24.28333E, 95 m, 03.v.2007, leg. L. Borowiec & M.L. Borowiec (DBET); 1w. (pin), Koutsomatados-Mili rd., 35.38333N,23.66666E, 308 m, 02.v.2011, leg. L. Borowiec (DBET); 2w. (EtOH), Omalos, 35.31667N,23.9E, 1122 m, 03.v.2014, leg. S. Salata (DBET); 1w. (pin), Plemeniana n. Kandanos, 35.31666N,23.71666E, 339 m, 02.v.2011, leg. L. Borowiec (DBET); 4w. (EtOH), Potamida n. Mythimna, 35.46666N,23.68333E, 37 m, 02.v.2011, leg. L. Borowiec (DBET); 17w. (EtOH), Therisso Gorge, 35.43333N,23.98333E, 320 m, 01.v.2011, leg. L. Borowiec (DBET); 3w. (EtOH), Ganies–Kalamaki road, 35.28333N,24.93333E, 439 m, 16.iv.2014, leg. S. Salata (DBET); 3w. (EtOH), Iraklion city-walls, 35.31667N,25.11667N, 46 m, 03.iv.2014, leg. S. Salata (DBET); 3w. (EtOH), Kastelli 1 km E, 35.2N,25,33333E, 363 m, 15.iv.2014, leg. S. Salata (DBET); 6w. (EtOH), Katofigi, 35.08333N,25.4E, 560 m, 12.iv.2014, leg. S. Salata (DBET); 2w. (EtOH), Miamou, 34.96667N,24.93333E, 494 m, 24.iv.2014, leg. S. Salata (DBET); 2w. (EtOH), Rouvas Forest loc. 1, 35.15N,24.93333, 1316 m, 10.iv.2014, leg. S. Salata (DBET); 3w. (EtOH), Rouvas Gorge, 35.13333N,24.9E, 455 m, 26.iv.2014, leg. S. Salata (DBET); 5w. (EtOH), Xanias–Miliarades road, 35.08333N,25.38333E, 504 m, 13.iv.2014, leg. S. Salata (DBET); 3w. (EtOH), Dead’s Gorge, 35.08333N,26.25E, 15 m, 09.iv.2014, leg. S. Salata (DBET); 3w. (EtOH), Gorge of Richtis, 35.16667N,25.98333E, 245 m, 08.iv.2014, leg. S. Salata (DBET); 4w. (EtOH), Hristos–Mathokotsana road, 35.08333N,25.56667E, 703 m, 11.iv.2014, leg. S. Salata (DBET); 3w. (EtOH), Kalami–Psari Forada road, 35.016667N,25.48333, 419 m, 12.iv.2014, leg. S. Salata (DBET); 5w. (EtOH), Kato Symi loc. 1, 35.05N,25.48333E, 1206 m, 12.iv.2014, leg. S. Salata (DBET); 5w. (EtOH), Lasithi Platou – Pinakiano, 35.18333N,25.45E, 806 m, 23.iv.2014, leg. S. Salata (DBET); 3w. (EtOH), Lastros, 35.13333N,25.88333E, 336 m, 06.iv.2014, leg. S. Salata (DBET); 10w. (EtOH), Limnakarou plato, 35.13333N,25.46667E, 1130 m, 26.iv.2014, leg. S. Salata (DBET); 1w. (EtOH), Merisini, 35.16667N,25.9333E, 309 m, 06.iv.2014, leg. S. Salata (DBET); 3w. (EtOH), Mesa Lasithi, 35.16667N,25.5E, 838 m, 28.iv.2014, leg. S. Salata (DBET); 2w. (EtOH), Moni Kapsa, 35.01667N,26.05E, 1 m, 10.iv.2014, leg. S. Salata (DBET); 3w. (EtOH), Neapoli–Vrisses road, 35.2333N,25.6E, 443 m, 05.iv.2014, leg. S. Salata (DBET); 2w. (EtOH), Perma–Koutounari road, 35.01667N,25.83333E, 0 m, 11.iv.2014, S. Salata (DBET); 2w. (EtOH), Schinokapsala–Agios Ioannis road, 35.05N,25.85, 452 m, 10.iv.2014, leg. S. Salata (DBET); 3w. (EtOH), Tourloti–Mirsini road, 35.15N,25.93333E, 266 m, 06.iv.2014, leg. S. Salata (DBET); 2w. (EtOH), Voila, 35.08333N,26.1E, 578 m, 10.iv.2014, leg. S. Salata (DBET); 12w. (EtOH), Ag. Joannis Forest loc. 2, 35.23333N,24.4E, 480 m, 06.v.2013, leg. S. Salata (DBET); 2w. (EtOH), Chromonastiri, 35.326944N,24.510278E, 262 m, 10.v.2013, leg. S. Salata (DBET); 1w. (EtOH), Katsifou Gorge, 35.2N,24.38333E, 57 m, 05.v.2013, leg. S. Salata (DBET); 5w. (EtOH), Kissos, 35.18333N,24.56667E, 623 m, 09.v.2013, leg. L. Borowiec (DBET); 1w. (pin), Klisidi, 35.26666N,24.63333E, 642 m, 06.v.2013, leg. L. Borowiec (DBET); 4w. (EtOH), n. Velonado, 35.25N,24.36667E, 373 m, 13.v.2013, leg. S. Salata (DBET); 1w. (EtOH), n. Vilandredo, 35.25N,24.31667E, 354 m, 13.v.2013, leg. S. Salata (DBET); 3w. (EtOH), Orthes Gorge, 35.33333N,24.68333, 318 m, 28.iv.2014, leg. S. Salata (DBET); 2w. (EtOH), Plakias, 35.191389N,24.395E, 4 m, 05.v.2013, leg. S. Salata (DBET); 41w. (EtOH), Plakias; Akrotiri Kakomouri, 35.16667N,24.398055E, 28 m, 05.v.2013, leg. S. Salata (DBET); 1w. (pin), road to Preveli Beach loc. 1, 35.16666N,24.45E, 58 m, 07.v.2013, leg. L. Borowiec (DBET); 1w. (pin), Spili, 35.21666N,24.53333E, 537 m, 09.v.2013, leg. L. Borowiec (DBET); 1w. (pin), Vistagi, 35.23333N,24.68333E, 563 m, 06.v.2013, leg. L. Borowiec (DBET); 1w. (pin), Xirokambos, 35.110556N,24.558889E, 24 m, 12.v.2013, leg. L. Borowiec (DBET); 3w. (EtOH), Kournas lake, 35.31667N,24.26667E, 30 m, 10.vii.1997, leg. P. Lymberakis (NHMC); 1w. (EtOH), Kardaki, 650 m, 35.2N,24.61667E, 21.vii.1999, leg. E. Nikolakakis (NHMC).

***Lasiuspsammophilus* Seifert, 1992**

**GREECE**, **Crete**: 4w. (EtOH), Agia Triada n. Kalamaki, 35.0508N,25.7542E, 1 m, 28.iv.2014, leg. S. Salata (DBET); 3w. (EtOH), Moni Kapsa, 35.01667N,26.05E, 1 m, 24.iv.2014, leg. S. Salata (DBET); 2w. (EtOH), Episkopi beach, 35.35N,24.35E, 0 m, 09.iv.2014, leg. S. Salata (DBET); 1w. (pin), Gerakari, 35.21666N,24.58333E, 751 m, 09.v.2013, leg. L. Borowiec (DBET); 1w. (pin), Plakias, 35.191389N,24.395E, 4 m, 05.v.2013, leg. L. Borowiec (DBET); 4w. (pin), Preveli Beach, 35.154444N,24.472778E, 10 m, 07.v.2013, leg. L. Borowiec (DBET); 1w. (EtOH), Irakleio, 35.33333N,25.13333E, 10 m, 18.ix.2010, leg. E. Panagiotou (NHMC); 2w. (EtOH), Chamaitoulo, 35.03333N,26.2E, 06.v.2001, 180 m, leg. E. Nikolakakis (NHMC).

***Lasiusturcicus* Santschi, 1921**

**GREECE**, **Crete**: 3w. (EtOH), Kalives river, 35.45N,24.13333E, 26 m, 03.v.2014, leg. S. Salata (DBET); 13w. (EtOH), Kato Daratso n. Chania, 35.5N,23.983333E, 35–40 m, 07.v.2011, leg. L. Borowiec (DBET); 4w. (pin), Koutsomatados–Mili rd., 35.38333N,23.66666E, 308 m, 02.v.2011, leg. L. Borowiec (DBET); 1w. (pin), Potamida n. Mythimna, 35.46666N,23.68333E, 37 m, 02.v.2011, leg. L. Borowiec (DBET); 1w. (pin), Agia Triada n. Kalamaki, 35.0508N,25.7542E, 1 m, 24.iv.2014, leg. S. Salata (DBET); 2w. (EtOH), Agios Eirini, 35.26667N,25.15E, 130 m, 02.iv.2014, leg. S. Salata (DBET); 4w. (EtOH), Avgeniki, 35.18333N,25.01667E, 227 m, 03.v.2014, leg. S. Salata (DBET); 3w. (EtOH), Kastelli 1 km E, 35.2N,25.33333E, 363 m, 16.iv.2014, leg. S. Salata (DBET); 5w. (EtOH), Katofigi, 35.08333N,25.4E, 560 m, 13.iv.2014, leg. S. Salata (DBET); 2w. (EtOH), Panastros, 35.11667N,24.98333E, 545 m, 03.v.2014, leg. S. Salata (DBET); 5w. (EtOH), Sfendili, 35.25N,25.38333E, 151 m, 06.iv.2014, leg. S. Salata (DBET); 2w. (EtOH), Stoli–Louves road, 35.03333N,25.01667E, 197 m, 30.iii.2014, leg. S. Salata (DBET); 2w. (EtOH), Xanias–Miliarades road, 35.08333N,25.38333E, 504 m, 25.iv.2014, leg. S. Salata (DBET); 3w. (EtOH), Dead’s Gorge, 35.08333N,26.25E, 15 m, 09.iv.2014, leg. S. Salata (DBET); 3w. (EtOH), Hametoulo, 35.05N,26.18333E, 520 m, 12.iv.2014, leg. S. Salata (DBET); 3w. (EtOH), Kalamafka, 35.06667N,25.65E, 472 m, 12.iv.2014, leg. S. Salata (DBET); 6w. (EtOH), Lasithi Platou – Pinakiano, 35.18333N,25.45E, 806 m, 28.iv.2014, leg. S. Salata (DBET); 2w. (EtOH), Praisos, 35.11667N,26.06667E, 193 m, 11.iv.2014, leg. S. Salata (DBET); 6w. (EtOH), Ag. Joannis forest loc. 1, 35.23333N,24.4E, 448 m, 06.v.2013, leg. S. Salata (DBET); 2w. (EtOH), Chromonastiri, 35.326944N,24.510278E, 262 m, 10.v.2013, leg. S. Salata (DBET); 3w. (EtOH), Frati, 35.2N,24.46666E, 297 m, 7.v.2013, leg. S. Salata (DBET); 2w. (EtOH), Kissos, 35.18333N,24.56667E, 623 m, 09.v.2013, leg. S. Salata (DBET); 1w. (EtOH), Kissou Kambros, 35.16666N,24.55E, 514 m, 14.v.2013, leg. S. Salata (DBET); 3w. (EtOH), Palelimnos, 35.3N,24.41666E, 262 m, 15.v.2013, leg. S. Salata (DBET); 4w. (EtOH), Plakias, 35.191389N,24.395E, 4 m, 05.v.2013, leg. S. Salata (DBET); 5w. (EtOH), road to Nida platou, 35.25N,24.88333E, 1166 m, 01.iv.2014, leg. S. Salata (DBET); 3w. (EtOH), Setoures, 35.26667N,24.38333E, 305 m, 15.v.2013, leg. S. Salata (DBET); 2w. (EtOH), Vistagi, 35.23333N,24.68333E, 563 m, 16.v.2013, leg. S. Salata (DBET); 1w. (EtOH), Agia lake, 35.46667N,23.93333E, 40 m, 08.vii.1997, leg. P. Lymberakis (NHMC); 2w. (EtOH), Almyros river, 35.33469N,25.05441E, 297 m, 03.vi.2012, leg. E. Aspradaki (NHMC); 1w. (EtOH), Aposelemis, 35.33333N,25.33333E, 7 m, 02.viii.2000, leg. A. Trichas (NHMC); 1w. (EtOH), Dikti mt., 35.11667N,25.46667, 1450 m, 05.viii.2000, leg. S. Simaiakis (NHMC); 2w. (EtOH), Dikti mt., 35.1N,25.46667E, 1750 m, 10.v.2001, leg. M. Chatzaki (NHMC); 2w. (EtOH), Before Amygdali after Neapoli, 35.2N,25.58333E, 561 m, 06.viii.1997, leg. I. Stathi (NHMC); 1w. (EtOH), Agios Titos, 35.18333N,24.75E, 1000 m, 14.iv.2000, leg. M. Papadimitrakis (NHMC); 1w. (EtOH), Ano Meros, 35.16667N,24.65E, 750 m, 15.iv.2000, leg. I. Stathi (NHMC); 3w. (EtOH), Garazo, 35.33333N,24.78333E, 100 m, 07.iv.2000, leg. E. Nikolakakis (NHMC); 1w. (EtOH), Tigania, 35.28333N,24.73333, 1100 m, 07.iv.2000, leg. E. Nikolakakis (NHMC).

**Figure 8.**
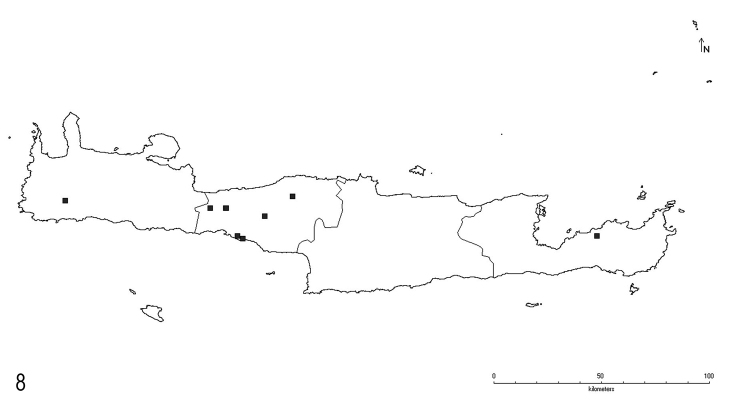
Distribution of *L.tapinomoides* sp. n. on Crete.

**Figure 9.**
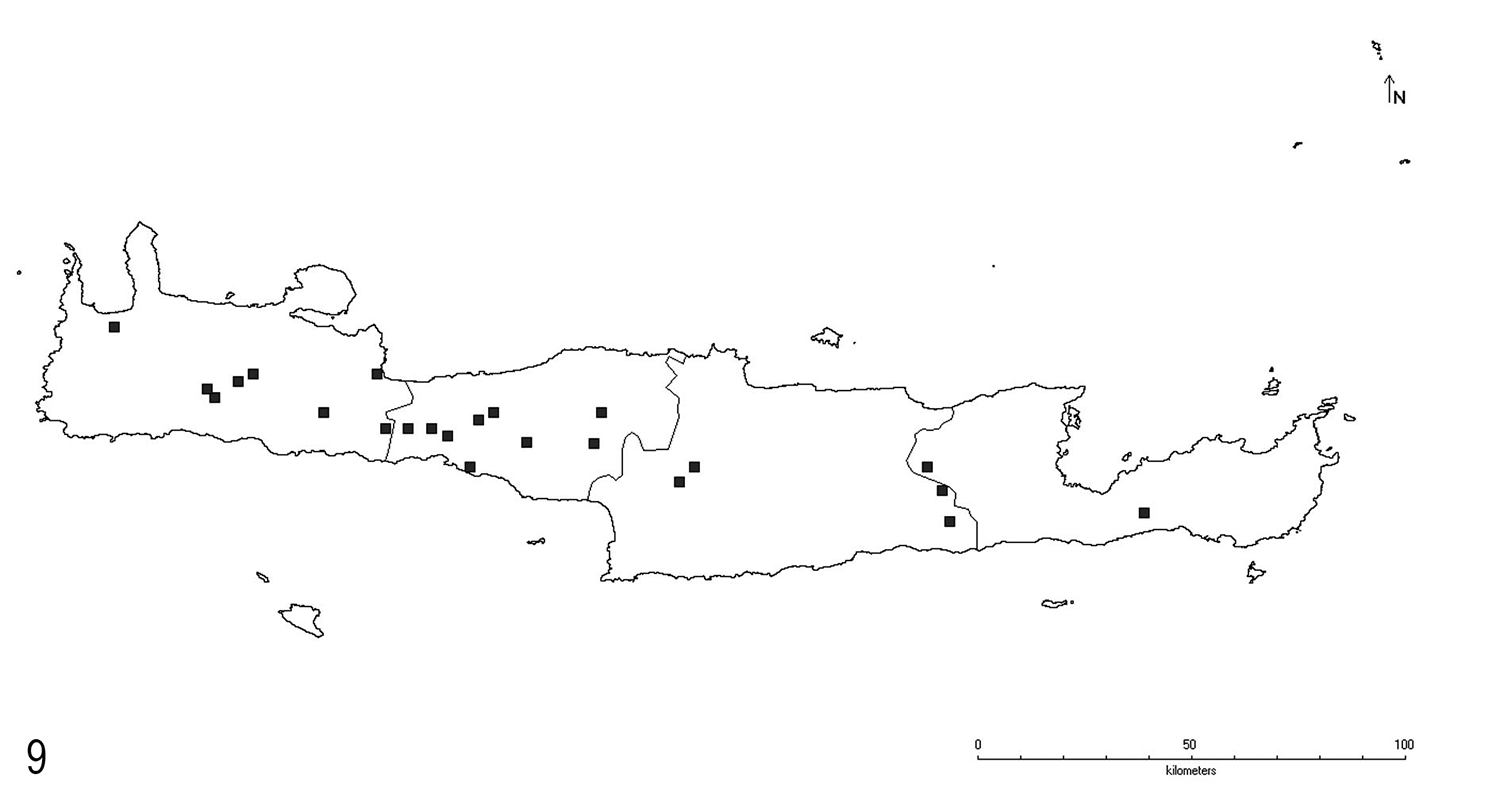
Distribution of *L.bombycina* Seifert & Galkowski on Crete.

**Figure 10.**
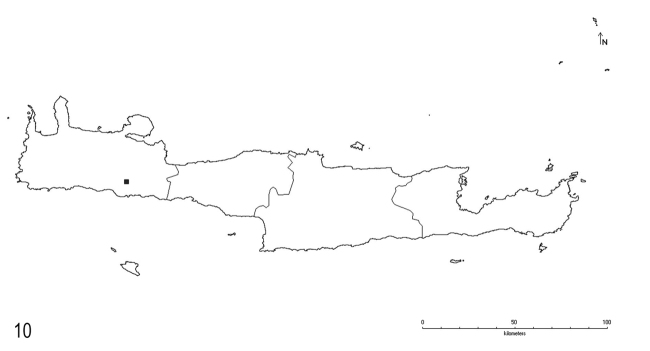
Distribution of *L.myops* Forel on Crete.

**Figure 11.**
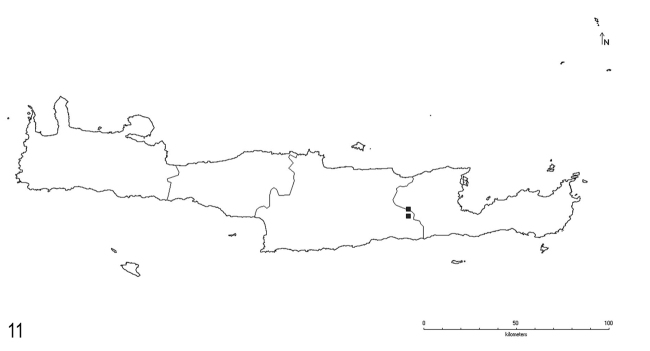
Distribution of *L.illyricus* Zimmerman on Crete.

**Figure 12.**
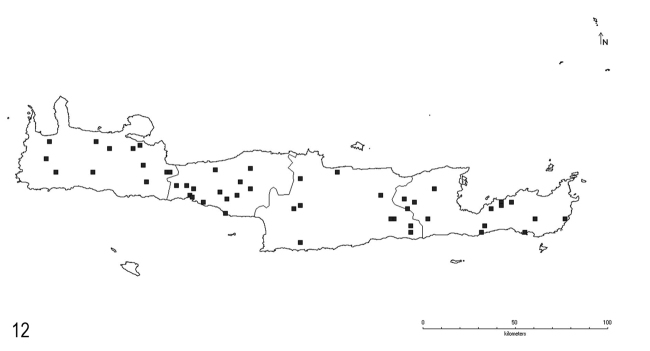
Distribution of *L.lasioides* (Emery) on Crete.

**Figure 13.**
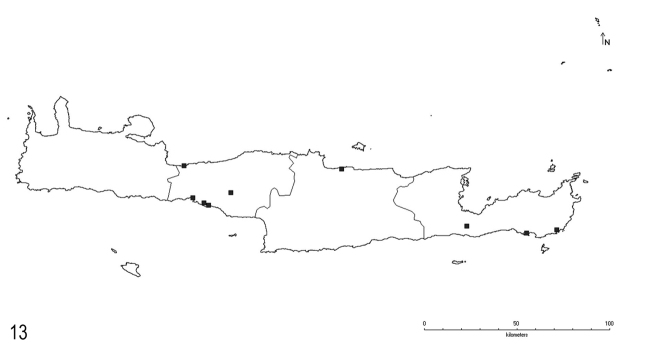
Distribution of *L.psammophilus* Seifert on Crete.

**Figure 14.**
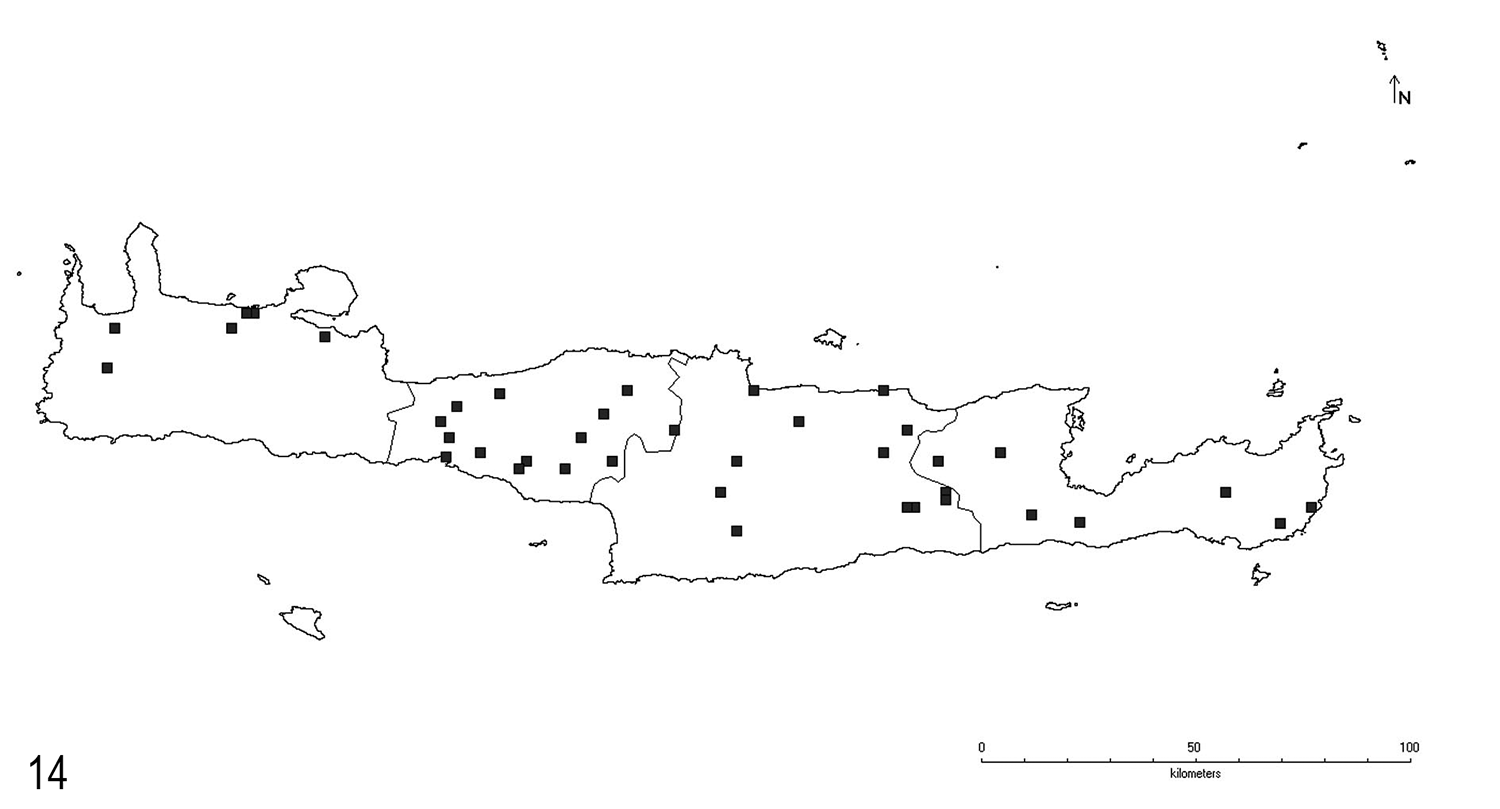
Distribution of *L.turcicus* Santschi on Crete.

**Figures 15–20.**
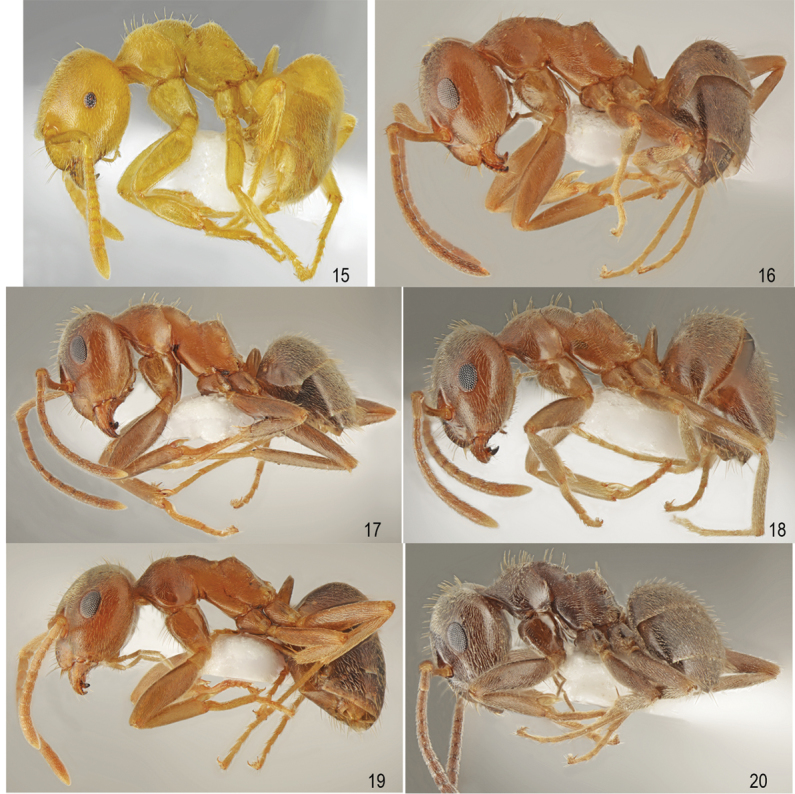
Lateral view. **15***Lasiusmyops* Forel **16***L.lasioides* (Emery) **17***L.illyricus* Zimmerman **18***L.psammophilus* Santschi **19***L.turcicus* Santschi **20***L.bombycina* Seifert & Galkowski.

**Figures 21–25.**
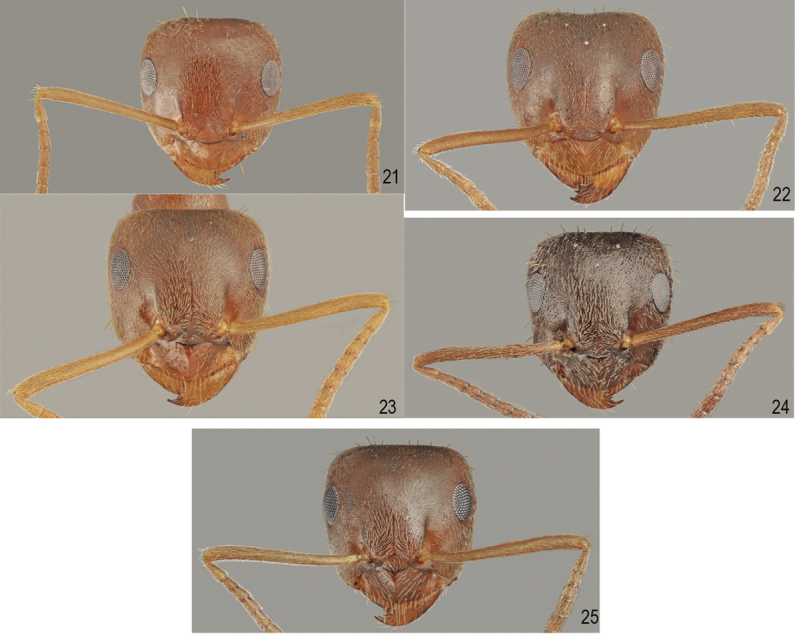
Head and scapus. **21***L.lasioides* (Emery) **22***L.illyricus* Zimmerman **23***L.turcicus* Santschi **24***L.bombycina* Seifert & Galkowski **25***L.psammophilus* Santschi.

##### Key to Cretan *Lasius* species (based on the worker caste)

**Table:** 

1.	Maxillary palps short, not reaching midpoint between mouth and occipital foramen, body yellow to orange (Fig. 15)	***L.myops* Forel**
–	Maxillary palps long, distinctly reaching beyond midpoint between mouth and occipital foramen, body brown to black or bicoloured (Figs 1, 16–20)	**2**
2.	Scape, genae and hind tibiae only with perfectly adpressed pubescence, without setae, occipital margin of head usually with 8 erect setae at most (Fig. 21)	***L.lasioides* (Emery)**
–	Scape, genae and hind tibiae often with occasional setae, pubescence not perfectly adpressed, occipital margin of head with more than 8 erect setae (Figs 22–25)	**3**
3.	Scape with few (>5) erect setae, body bicoloured, hind tibiae with numerous erect setae, mesosoma brighter than head and gaster (Figs 17, 22)	***L.illyricus* Zimmerman**
–	Scape without or with maximum 5 erect setae, hind tibiae without or with a few erect setae, body uniformly coloured or head and mesosoma uniformly coloured, brighter than gaster (Figs 18–20, 23–25)	**4**
4.	Clypeus with dense pubescence, average distance between setae 3.5 times shorter than their length (Fig. 24)	***L.bombycina* Seifert & Galkowski**
–	Clypeus with sparse pubescence, average distance between setae equal or longer than a half of their length (Figs 3–4, 23, 25)	**5**
5	Workers small, ML 0.726–0.827 mm, mesosoma with very shallow metanotal groove, apical part of scape with >5 suberect to erect setae (Figs 1–4)	***L.tapinomoides* sp. n.**
–	Workers larger, ML 0.935–1.18 mm, metanotal groove distinct, apical part of scape without or with <5 suberect to erect setae (Figs 18–19, 23, 25)	**6**
6	Metanotal groove relatively shallow, propodeal dorsum flattened, hind tibia usually without suberect setae, number of mandibular dents < 7.7 (Fig. 19)	***L.turcicus* Santschi**
–	Metanotal groove deeper and sharp, propodeal dorsum convex, hind tibia with few suberect setae, number of mandibular dents >7.7 (Fig. 18)	***L.psammophilus* Santschi**

## Discussion

Based on the literature (Forel 1886, 1889, 1910, Neuenschwander et al.1983, Legakis 2011, Borowiec and Salata 2012) there are 7 *Lasius* species reported from Crete: *L.alienus*, *L.brunneus* (Latreille), *L.niger* (Linnaeus), *L.paralienus* Seifert, *L.lasioides*, *L.psammophilus*, and *L.turcicus*, all members of the subgenus Lasius s. str. Our study confirmed the presence of the last 3 listed species and shown 4 species new for Cretan fauna: *L.bombycina*, *L.myops*, *L.illyricus* and *L.tapinomoides*, the latter being endemic to Crete and new to science. The first records of *L.alienus* and *L.niger* come from literature published in the 19^th^ century (Forel 1886, 1889). Seifert (1992) proved that Crete is beyond the range of occurrence of these species, thus those records probably refer to *L.bombycina* or *L.psammophilus*. Based on most recent revisions (Seifert 1992, Seifert and Galkowski 2016) *Lasiusbrunneus* and *L.paralienus* should be also excluded from the list of Cretan species. On Crete they are replaced by *L.lasioides* and *L.bombycina* respectively. *Lasiusmyops* and *L.illyricus* were recorded on Crete only from high mountains (above 1000 m a.s.l.). These two species are common on Greek mainland, and most often were recorded from lower altitudes. Cretan record of *L.illyricus* is so far the southernmost known place of occurrence of this species. It is worth noting that all *Lasius* species known from Crete manifest independent colony foundation. The updated list of Cretan *Lasius* is as follows: *L.bombycina*, *L.myops*, *L.illyricus*, *L.lasioides*, *L.psammophilus*, *L.tapinomoides*, and *L.turcicus*, six members of *Lasius* s. str. and one representative of *Cautolasius*.

## Supplementary Material

XML Treatment for Lasius tapinomoides
